# Proximity labeling to detect RNA–protein interactions in live cells

**DOI:** 10.1002/2211-5463.12706

**Published:** 2019-09-25

**Authors:** Mingxing Lu, Wencheng Wei

**Affiliations:** ^1^ Department of Biomedical Sciences College of Veterinary Medicine and Life Sciences City University of Hong Kong Kowloon Tong Hong Kong; ^2^ Department of Biology Southern University of Science and Technology Shenzhen China

**Keywords:** APEX2, birA*, nascent peptide, proximity labeling, RNA binding protein, RNA–protein interactions

## Abstract

RNA biology is orchestrated by the dynamic interactions of RNAs and RNA‐binding proteins (RBPs). In the present study, we describe a new method of proximity‐dependent protein labeling to detect RNA–protein interactions [RNA‐bound protein proximity labeling (RBPL)]. We selected the well‐studied RNA‐binding protein PUF to examine the current proximity labeling enzymes birA* and APEX2. A new version of birA*, BASU, was used to validate that the PUF protein binds its RNA motif. We further optimized the RBPL labeling system using an inducible expression system. The RBPL (λN‐BASU) labeling experiments exhibited high signal‐to‐noise ratios. We subsequently determined that RBPL (λN‐BASU) is more suitable than RBPL (λN‐APEX2) for the detection of RNA–protein interactions in live cells. Interestingly, our results also reveal that proximity labeling is probably capable of biotinylating proximate nascent peptide.

**Abbreviations**
APEX: ascorbate peroxidase
BASU: birA*, from *Bacillus subtilis*
bioID: proximity‐dependent biotin identification
CLIP: cross‐linking immunoprecipitation
RAP: RNA antisense purification
RBPL: RNA‐bound protein proximity labeling
RBPs: RNA‐binding proteins
RIP: RNA immunoprecipitation

RNA–protein interactions are pervasive in cells 1, 2. RNAs serve as the binding sites for RBPs to form ribonucleoprotein. RNAs function together with specific binding proteins to determine post‐transcriptional processes, including translation, RNA splicing, cleavage and polyadenylation, RNA editing, RNA localization and decay 3, 4, 5, 6, 7, 8, 9. RNA–protein interactions execute numerous roles in cellular functions and diseases. There are diverse methods for detecting and characterizing RNA–protein interactions 2, 10, 11, 12. One method of RNA antisense purification (RAP) is to isolate specific long non‐coding RNA and its associated proteins 13, 14, 15. These associated proteins are then identified by quantitative MS. Further validation and characteristics are performed. In the RAP‐MS method, UV cross‐linking is used to generate covalent bonds between the contacting RNA and protein, purifying RNAs in denaturing conditions to eliminate non‐specific interactions. Such attempts have sufficiently defined the roles of Xist‐mediated transcriptional silencing via direct interaction with protein complexes in X‐chromosome inactivation 14. RAP‐MS is an applicable method for the biochemical isolation of RNA‐binding proteins by enriching specific RNA and its associated RNA complexes from native cell lysis. However, this method only identifies highly abundant RNAs and isolates interactions that are cross‐linked in cells. It requires a huge quantity of starting materials to obtain high purification yields of RNA–protein complexes because any individual RNA is probably present at only a very small proportion of the total cellular RNAs.

Chromatin immunoprecipitation has been used to determine chromatin bound DNA motifs. Similarly, immunoprecipitation‐derived methods have also been proposed for the detection of RBPs, termed RNA immunoprecipitation (RIP) 16, 17, 18, 19, 20, 21. RIP involves the immunoprecipitation of an RBP of interest, which employs a specific antibody. The RNAs that co‐immunoprecipitated with the protein are then subjected to sequence for identification. This method is referred to as RIP‐seq. If UV cross‐linking is applied, it would derive as cross‐linking immunoprecipitation (CLIP) 22. In cells, RNAs are always complexed with RBPs, whereas UV light of wavelength 254 nm (i.e. UV‐C) induces covalent bonds between complexes of RBPs and their contacting RNAs. The RBP–RNA complexes could be enriched with a specific antibody, and bound RNAs are eluted from the RBP–RNA complexes and then determined. This procedure is relatively inefficient and time‐consuming. RIP‐seq and CLIP‐seq are important methods for studying RNA–protein interactions. The major concerns are the efficiency of cross‐linking and the availability of specific antibodies. In CLIP‐seq, UV cross‐linking is more specific, although it only links proteins to RNAs that are at near‐zero distance 23.

The complexity of RNA–protein interaction in specific cellular context is incompletely addressed. Therefore, an efficient detection method for studying RNA–protein interactions is critical to a full understanding of gene expression regulation. Recently, Ramanathan *et al*. 24 utilized proximity‐dependent protein labeling to efficiently identify the proteins that bind RNA transcript of interest in intact live cells. Proximity‐dependent labeling employs enzymes that produce reactive radicals to covalently tether proximate proteins with biotin. The biotinylated proteins can then be purified in a denatured condition for further analysis by western blotting or MS. Based on the labeling enzymes used to catalyze reaction, proximity‐dependent labeling methods can be classified into two mainstreams: engineered ascorbate peroxidase (APEX or APEX2) and bacterial biotin ligase mutant, birA* (bioID or bioID2). APEX uses biotin‐phenol as substrates, and H_2_O_2_ is required to ignite the enzyme reaction. APEX catalyzes biotin‐phenol into biotin‐phenoxyl radicals that diffuse to the surrounding milieu and react with proximate proteins in electron‐rich amino acid side chains. BirA* uses biotin as substrates to produce biotinoyl‐5ꞌ‐AMP radicals, which then react with lysine residues on proximate proteins.

Ramanathan *et al*. 24 engineered a derived promiscuous biotin ligase enzyme, birA*, from *Bacillus subtilis*, termed BASU. BASU rapidly biotinylates proteins bound to specific RNA motifs; biotinylated proteins are then separated by streptavidin pulldown, followed by MS to identify RNA interacting proteins. We tested BASU in the detection of RNA–protein interaction by transient transfection. We initially chose a well‐known RNA–protein interaction pair: PUF and PUF binding motif 25, 26. This is because the PUF protein recognizes its target RNA motif in a modular manner and binds it with high affinity (*K*_d_ ≈ 18 nm). Subsequently, we generated an inducible stable cell line of BASU for labeling efficiency and consistency.

To date, birA* and APEX2 have been successfully applied to study a variety of proteins and processes in cells 27, 28, 29, 30, 31, 32, 33. APEX2 labels proximate proteins within 1 min. APEX2 is probably the most active proximal labeling enzyme, which makes it very suitable for capturing dynamic processes. Two recent studies have showed the utilization of APEX2 in capturing the ‘snapshot’ of proximate interactions in a fast signaling turnover 27, 28. The technique also works well in confined compartments 29, 30. The prompts the question of whether APEX2 might also be suitable for RNA–protein interaction detection. In the present study, we aimed to establish an optimal RNA‐bound protein proximity labeling (RBPL) method for identifying RNA–protein interactions in live cells. We addressed this question by performing a side‐by‐side comparison of two proximity labeling enzymes, BASU and APEX2, in which we utilized PUF as a point of reference. Stable expression system was applied to overcome the bias of transient transfection.

## Results

### Design of RBPL

**Figure 1:**
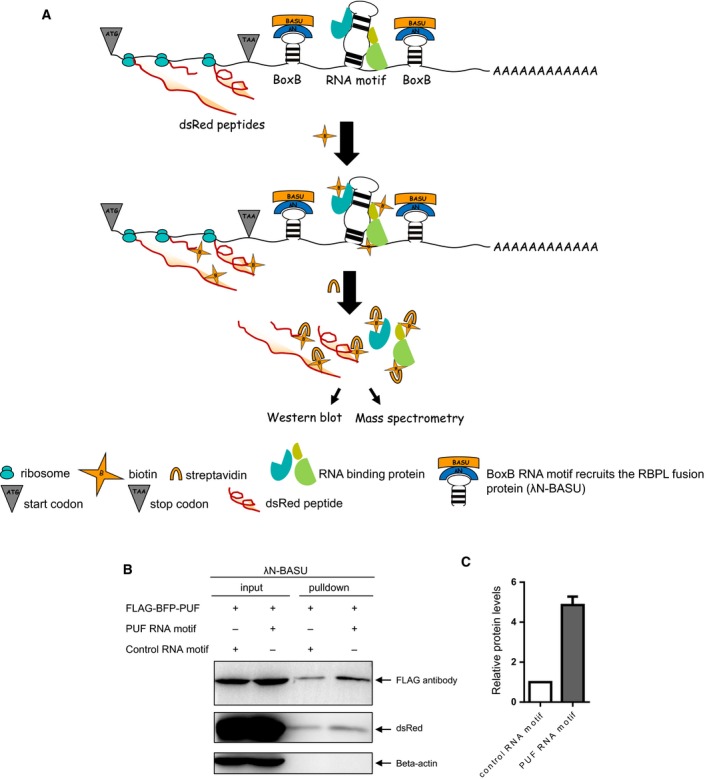
RNA‐bound protein proximity labeling (RBPL). (A) Schematic representation of RBPL. BoxB RNA motif sequences flank the RNA motif of interest. BoxB RNA motif recruits the RBPL fusion protein (λN‐BASU), leading to the biotinylation of proteins proximal to the inserted RNA motif in live cells. Biotinylated proteins are then separated by streptavidin pull down, followed by western blotting or MS analysis. (B) Validation of RBPL with PUF RNA motif‐bound proteins in transient transfection of HEK‐293T cells. RBPL biotinylates proteins proximal to inserted PUF RNA motif, PUF proteins and nearby dsRed peptides. Beta‐actin proteins are not biotinylated. (C) Semi‐quantitative analysis of biotinylated PUF proteins by RBPL on the PUF RNA motif and scrambled control RNA motif. Error bar represents the mean ± SD derived from three independent experiments.

RBPL comprises two elements: an RNA element and a RBPL protein. The RNA element is composed of a BoxB RNA motif flanking any RNA motif of interest. The 22‐amino‐acid λN peptide recognizes BoxB RNA motif at high affinity; the λN peptide fused to the N terminus of the BASU biotin ligase (λN‐BASU) comprises the protein component. BoxB RNA motif recruits the RBPL protein, thereby biotinylating proteins bound to the flanked adjacent RNA motif of interest (Fig. 1A), allowing capture by streptavidin of RNA motif‐bound proteins for analysis by western blotting or MS. Western blotting confirms RNA‐bound proteins, whereas MS identifies unknown RNA‐bound proteins.

### Validation of RBPL with a known RNA–protein interaction

PUF is a modular RNA‐binding protein. It binds specifically to UUGAUAUA RNA motif at high affinity 25, 26. BoxB RNA motif sequences flank the PUF RNA motif. BoxB RNA motif recruits the RBPL fusion protein (λN‐BASU), leading to the biotinylation of proteins proximal to the flanked PUF RNA motif, flag‐BFP‐PUF protein and peptide dsRed in the vicinity (Fig. 1B). Next, we considered whether the RBPL biotinylation has labeling resolution in cells because proximity labeling is based on the diffusion of reactive radicals. Beta‐actin, known as a housekeeping protein, is constitutively and stably expressed at high levels in cells. As we show, beta‐actin proteins are not biotinylated in this process (Fig. 1B). We performed semi‐quantification of biotinylated PUF proteins by RBPL on PUF RNA motifs and scrambled controls. The RBPL with PUF RNA motif yielded an approximately five‐fold enrichment of PUF proteins over scrambled controls in transient transfection of HEK‐293T cells (Fig. 1C).

### Generation of stable expression of RBPL cell lines

**Figure 2:**
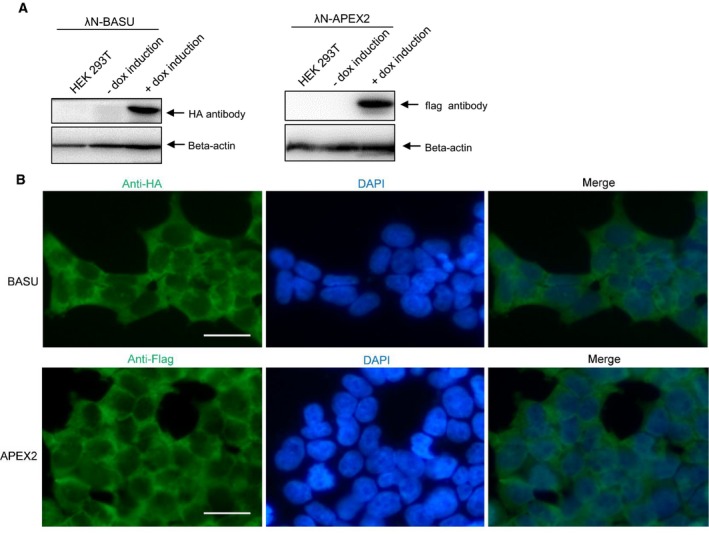
Generation of stable expression of RBPL cell lines. (A) The stable expression of RBPL was generated under the inducible control of a Tet‐On system in HEK‐293T cells. After dox induction, the inducible expression of RBPL (λN‐BASU) and RBPL (λN‐APEX2) was confirmed by western blotting. (B) RBPL expression was further checked with an immunofluorescent cytochemical staining assay. Fluorescence microscopy analysis reveals that the cellular expression of RBPL is mainly in the cell cytosol. Scale bar = 25 μm. HEK‐293T cells were seeded on pre‐coated coverslips. After dox induction for 24 h, cells were fixed and stained. The cellular localization was visualized by immunofluorescence staining using Alexa Fluor 488 (green). Cell nuclei stained with DAPI (blue). RBPL (λN‐BASU) was detected with anti‐HA. RBPL (λN‐APEX2) was detected with anti‐flag.

Although previous studies have reported that proximity‐dependent labeling has a high background in mammalian cells 27, 28, 34, 35, we have achieved a very good signal‐to‐noise ratio in this transient transfection experiment. Proximity labeling biotinylates proteins in a way that relies on proximal distance and duration time. We have further optimized this proximity labeling procedure by generating stable cell lines (Fig. 2).

We generated a stable expression of RBPL under the inducible control of a Tet‐On system in HEK‐293T cells. The inducible expression of RBPL (λN‐BASU) was confirmed by western blotting, as well as RBPL (λN‐APEX2) expression (Fig. 2A). The biotinylation function of RBPL can be fine‐tuned with tetracycline‐inducible expression. We also determined the RBPL expression with an immunofluorescent cytochemical staining assay. The results obtained reveal that the RBPL is mainly expressed in the cell cytosol (Fig. 2B).

### Comparison of RBPL (λN‐BASU) and RBPL (λN‐APEX2) in RNA–protein interaction detection in live cells

**Figure 3:**
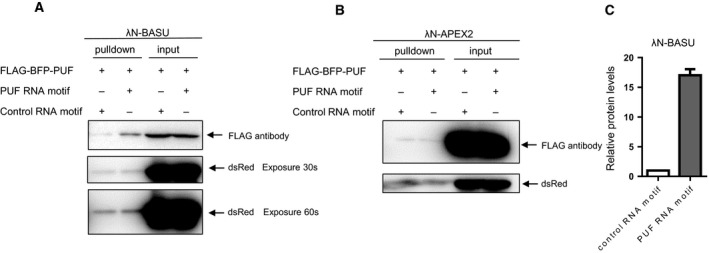
RBPL (λN‐BASU) and RBPL (λN‐APEX2) in RNA–protein interaction detection. (A) Proximity labeling by RBPL (λN‐BASU) on the PUF RNA motif and scrambled control RNA motif. The biotinylation labeling by RBPL showed an excellent signal‐to‐noise ratio via the generation of stable cell lines. (B) Proximity labeling by RBPL (λN‐APEX2) on the PUF RNA motif and scrambled control RNA motif. The RBPL (λN‐APEX2) biotinylation labeling is very weak, with almost no discernible signal between the PUF RNA motif and scrambled controls. (C) Semi‐quantitative analysis of biotinylated PUF proteins by RBPL (λN‐BASU) on the PUF RNA motif and scrambled control RNA motif. The RBPL (λN‐BASU) with PUF RNA motif yielded an approximately 17‐fold enrichment of PUF proteins over scrambled controls. Error bar represents the mean ± SD derived from three independent experiments.

The RBPL biotinylation labeling was controlled under the tetracycline induction. The RBPL (λN‐BASU) with PUF RNA motif yielded an approximately 17‐fold enrichment of PUF proteins over scrambled controls (Fig. 3A,C). The biotinylation labeling by RBPL showed excellent signal‐to‐noise ratio via the generation of stable cell lines. However, the RBPL (λN‐APEX2) biotinylation labeling is very weak, with almost no discernible signal between PUF RNA motif and scrambled controls (Fig. 3B). In comparison, RBPL (λN‐BASU) is more suitable than RBPL (λN‐APEX2) for the detection of the RNA–protein interaction in live cells.

## Discussion

In the present study, we describe RBPL as a method for detecting RNA–protein interactions in live cells. RBPL labels proteins that bound to specific RNA motif with biotin in a cellular environment, which could overcome bias from artificial RNA–protein interactions or post‐lysis interactions. RBPL biotinylates proximal proteins within a range of reactive radical diffusion. The biotinylation of proximal proteins by RBPL allows for stringent washing to identify true proximal proteins. Potentially, RBPL permits the study of specific RNA foci associated proteomes in live cells when combined with quantitative proteomics.

Previous work has reported that the detection of RNA–protein interaction only uses transient transfection 24. Three plasmids were transiently transfected within one culture plate at a time and it was claimed that exceptional signal‐to‐noise ratio and fast kinetics had been achieved. In the present study, we suspect that this may be highly dependent on efficient transfection or the specific context because proximity‐dependent labeling was only effective under the situation when three plasmids simultaneously entered the cells. With transient transfection, this is not reproducible in our experience. We optimized the RBPL method to obtain an improved signal‐to‐noise ratio by generating a stable cell line. The inducibility of RBPL provides temporal expression for controlling the total docking time during which the labeling enzyme occupies the targeted RNA foci and minimizes the accumulation of excessive labeling activity. In the labeling results readout, we used biotinylated dsRed as an internal control instead of labeling enzyme expression because the dsRed located nearby could be a perfect labeling activity indicator of proximity labeling enzyme and is distributed all over the cell (Fig. S1). This may help to avoid unforeseeable system errors. In addition, our results reveal that proximity labeling by RBPL is capable of biotinylating peptides in its vicinity, as detected by dsRed antibody. These nearby peptides are probably newly synthesized.

The method presented here has some limitations in its current form. For example, RBPL is unable to distinguish between directly protein‐binding or indirectly proteins associated with RNA. Moreover, birA* or its derived variant‐based proximity labeling biotinylates primary amines of lysines 32, 36. It may not provide an unbiased detection chance as a result of unequal lysine residues appearing on the outer surface of proximate proteins. However, APEX2 catalyzes biotin‐phenols into reactive biotin‐phenoxyl radicals that diffuse to the surrounding milieu and react with proximal proteins in electron‐rich amino acid side chains. Thus, we also tested the APEX2‐based RBPL method.

However, APEX2‐based labeling suffers from excessive background biotinylation that is unbound to the target RNA motif. Almost no discernible signal is seen between the PUF RNA motifs and the scrambled controls. Based on what we have found in the literature, APEX2 can identify proximate associations, although only with appropriate controls (not unlike birA*) and with SILAC labeling (unnecessary for birA*) 37, 38, 39, 40. BirA* is the predominant method used to identify candidate protein–protein interactions 32, 34, 35, 41. APEX2 has predominantly been used for compartmental proteomics, although, if used properly, it can be successful for identifying candidate protein–protein interactions or RNA–protein interactions (Dr Kyle Roux, personal communication). BirA* is a slow enzyme, and so perhaps even if it has a longer half‐life *in vitro*, it is unable to saturate its ‘target rich’ environment because the low levels of reactive biotin are unable to travel far before reacting with something, regardless of whether this is a protein, a free amino acid or another reactive molecule in the crowded cellular environment. APEX2, on the other hand, generates a massive amount of reactive biotin in a short period of time, thus potentially saturating the proximate environment of reactive molecules and enabling more distal labeling.

Regarding the differential stability of the radicals from birA* and APEX2, we have not identified any compelling comparative *in vivo* evidence regarding this in the literature. The present study describes, for the first time, a side‐by‐side comparison of the two enzymes in the detection of RNA–protein interaction in the cellular environment.

## Materials and methods

### Molecular cloning

pSpCas9(BB)‐2A‐GFP (PX458) was a generous gift from Feng Zhang (Addgene plasmid #48138). To build the PUF protein expression plasmid, the PX458‐FLAG‐BFP‐PUF plasmid was generated by replacing the SpCas9(BB)‐2A‐GFP cassette in PX458 with the FLAG‐BFP‐PUF fragment. The puf DNA fragment was PCR amplified from pGL‐CPSF1‐PUF (67‐2) vector (a gift from the laboratory of Dr Zefeng Wang in CAS‐MPG Partner Institute for Computational Biology, Shanghai, China), generated FLAG‐BFP‐PUF fragment by overlapping PCR and subcloned into PX458 by homologous recombination in accordance with the manufacturer's instructions (ClonExpress MultiS One Step Cloning Kit; Vazyme Biotech Co.,Ltd, Nanjing, China). To build the RNA motif expression plasmid, pRiG‐SV40‐3X BoxB‐5X puf‐3X Box B, 5X puf motif seq and 3X Box B motif seq were synthesized (GENEWIZ, Inc., Bishop's Stortford, UK): Box B seq: GCCCTGAAAAAGGGC. 5X puf seq:

TTGATATAGGTTCGGTTGATATAGGGTTGATATAGGGTTGATATACGGTTGATATA. 5X puf seq was located between two 3X Box B seq, generated by overlapping PCR and subcloned into pRiG‐SV40 vector non‐translated region behind dsRed. pRiG‐SV40 vector was a gift from the laboratory of Dr Bin Tian in Rutgers New Jersey Medical School, Newark, NJ, USA. PUF protein cannot recognize base cytosine. Scrambled RNA sequence containing cytosine could be used as a control RNA motif. pRiG‐SV40‐3X BoxB‐5X scrambled control‐3X BoxB plasmid was constructed similarly. Scrambled control seq:

AGGTAAACCCCAGGTAAACCCCAGGTAAACCCCAGGTAAACCCCAGGTAAAC. pcDNA3.1‐Hygro(+) is commonly used for transient transfection studies. To generate BASU labeling plasmid pcDNA3.1‐Hygro(+)‐HA‐λN‐BASU, BASU seq was codon‐optimized for mammalian cell expression and synthesized, a 66‐bp λN (BoxB binding protein) sequence was synthesized, and HA‐λN‐BASU fragment was generated by overlapping PCR and subcloned into pcDNA3.1‐Hygro (+) vector between the *Bam*H1 and *Eco*RV sites.

RAR3G‐APEX2‐FLAG plasmid 42 (from the laboratory of Dr Ruijun Tian in Sust, Shenzhen, China) expresses APEX2‐flag, containing Tet‐on doxycycline‐inducible expression vector, and could be used for lentivirus infection and packaging for the generation of stable cell line. To construct APEX2 labeling plasmid RAR3G‐APEX2‐FLAG‐λN, a 66‐bp λN (BoxB binding protein) sequence was codon‐optimized for mammalian cell expression and synthesized, introduced by homologous recombination according to the manufacturer's instructions (ClonExpress II One Step Cloning Kit; Vazyme). To construct RAR3G‐HA‐λN‐BASU, HA‐λN‐BASU fragment was codon‐optimized and synthesized. RAR3G‐APEX2‐FLAG plasmid was digested into RAR‐3G with *Bam*H1 and *Pac*I, then recombined with HA‐λN‐BASU fragment, generating RAR3G‐HA‐λN‐BASU.

### Cell culture and transfection

HEK‐293T cells were maintained in Dulbecco's modified Eagle's medium supplemented with 10% FBS, cultured at 37 °C with 5% CO_2_ in an incubator. For transfection, cells were seeded at 40–60% confluence; the next day, the DNA and Lipofectamine 2000 (Life Technologies, Grand Island, NY, USA) were mixed and added to the seeding cells in accordance with the manufacturer's instructions.

### Generation of stable cell lines

HEK‐293T cells were grown in Dulbecco's modified Eagle's medium. RAR3G‐APEX2‐FLAG was a gift as transfer plasmid from the laboratory of Dr Ruijun Tian in Sust, Shenzhen, China. RAR3G‐APEX2‐FLAG expresses the Tet‐On 3G‐T2A‐PuroR protein constitutively from an EF1 alpha promoter. To generate RBPL (λN‐BASU) and RBPL (λN‐APEX2) inducible 293T stable cell line, we subcloned λN‐BASU‐HA, λN‐APEX2‐FLAG into the vector of the Tet‐on expression system, respectively. The Tet‐On system expresses high levels of target gene only when cultured in the presence of doxycycline (dox), a tetracycline analog. For the assembly of package lentivirus, 293T cells were packaged with the Addgene lentivirus production protocol (Addgene, Cambridge, MA, USA). Plasmid was packaged into lentivirus and then transfected into 293T cells. Forty‐eight hours after transfection, cells were selected with 2 μg·mL^−1^ puromycin in the culture media for 2 weeks. When transferred to the new plate, puro treatment was immediately added. Then, 1 μg·mL^−1^ doxycycline was added to the cell culture for expression induction. The expression of APEX2 and BASU cells were confirmed by western blotting.

### Proximity labeling in live cells

BASU labeling was performed in live cells. BASU is a new derived variant of promiscuous biotin ligase mutant, which uses biotin as substrate to generate radicals in live cells. Biotin labeling was performed as described previously 24. Labeling duration was modified to 2 h. For APEX2 labeling in live cells, APEX2 catalyzed biotin‐phenol as substrate into reactive radicals  with the addition of H_2_O_2_. Biotin‐phenol (BP) labeling was performed as described previously 29, 30. Plasmids were transfected into HEK293T cells with Lipofectamine 2000.

For transient transfection, 293T cells were analyzed 24 h after transient transfection with respect to labeling enzymes BASU‐N plasmid and PUF protein expression plasmid, along with plasmid containing RNA motif. In BASU stable 293T cells, transfect PUF protein expression plasmid and RNA motif expression plasmid. RNA transcripts contain PUF binding sites. Proximity labeling enzymes BASU biotinylate proteins that bind to these RNA transcripts, and streptavidin‐beads pull down biotinylated proteins. PUF was detected by its fusion flag. The APEX2 transfection procedure is the same as BASU. After 24 h of cell culture, BP was added to the culture media for 30 min. The medium was prewarmed to 37 °C to facilitate BP dissolution. Thirty minutes later, H_2_O_2_ was added at a final concentration of 1 mm, and the cells were incubated at room temperature for 1 min. Then, the labeling medium solution was quickly discarded and the reaction was stopped with cold quenching buffer (10 mm sodium ascorbate, 10 mm sodium azide and 5 mm Trolox). Cells were washed twice with quenching buffer, followed by three washes with PBS. Cells were then scraped onto the plate with 1 mL of RIPA buffer and detected by western blotting or MS.

### Cell lysis and western blot analysis of proximity labeling

Cell pellets were lysed by RIPA lysis buffer [50 mm Tris, 150 mm NaCl, 1% Triton X‐100, 0.5% sodium deoxycholate, 0.1% SDS, 1 × protease cocktail (catalog no. 4693159001; Roche Basel Switzerland) and 1 mm phenylmethanesulfonyl fluoride]. This was followed by incubation on ice for 10 min. Lysates were clarified by centrifugation at 21 100 ***g*** for 10 min at 4 °C. The supernatant was collected. Then protein concentration was measured using the BCA Protein Assay Kit (catalog no. P0011; Beyotime Biotech Co.,Ltd, Jiangsu, China). Sample processing and the pull‐down procedure are described in a previous study 24. Protein samples were separated on a 12% SDS/PAGE gel, 0.22 μm transfer membrane, 1.5 μm gel thick (100 V for 2 hours). Antibodies used in this study, anti‐flag (dilution 1:1000; (Sigma, St Louis, MO, USA), anti‐dsRed (dilution 1:1000; Abbkine, Hubei, China).

### Immunofluorescent staining

Cells were grown on coated coverslips. After dox induction for 24 h, cells were washed with ice‐cold PBS and fixed with 4% paraformaldehyde for 15 min at room temperature. They were then washed three times followed by permeabilization with 0.5% Triton X‐100 in PBS (PBST) for 5 min. Next, they were blocked for 30 min with 3% BSA in PBS at room temperature or at 4 °C overnight. The APEX2 was detected with flag antibody, whereas BASU was detected with anti‐HA. All primary antibodies were diluted 1 : 100 and incubated overnight at 4 °C. After three washes with PBS, cells were incubated with secondary Alexa Fluor 488‐conjugated Affinipure Goat Anti‐Mouse IgG(H+L) for 1 h at room temperature. Secondary antibody was used at a dilution of 1 : 3000. Cells were washed three times with PBST. Cell‐containing coverslips were mounted using a small drop of mounting medium with 4′,6‐diamidino‐2‐phenylindole. Examinations were performed by fluorescence microscopy (Ti‐E; NiKon, Tokyo, Japan).

## Conflict of interest

The authors declare that they have no conflict of interest.

## Author contributions

ML designed and performed the experiments. WC provided suggestions and comments. LF provided technological advice. WW and ML curated the data. ML wrote the manuscript.

## Supporting information


**Fig. S1.** Transient transfection of dsRed in HEK‐293T cells. (A) dsRed expressed all over the cell; (B) in DIC. Scale bar = 50 μm.
