# Supplementary figures and images for: Proximity labeling to detect RNA–protein interactions in live cells
Source: FEBS Open Bio. 2019 Sep 25;9(11):1860–8. doi: 10.1002/2211-5463.12706 (PMC6823345; doi:10.1002/2211-5463.12706)

Fig. S1A

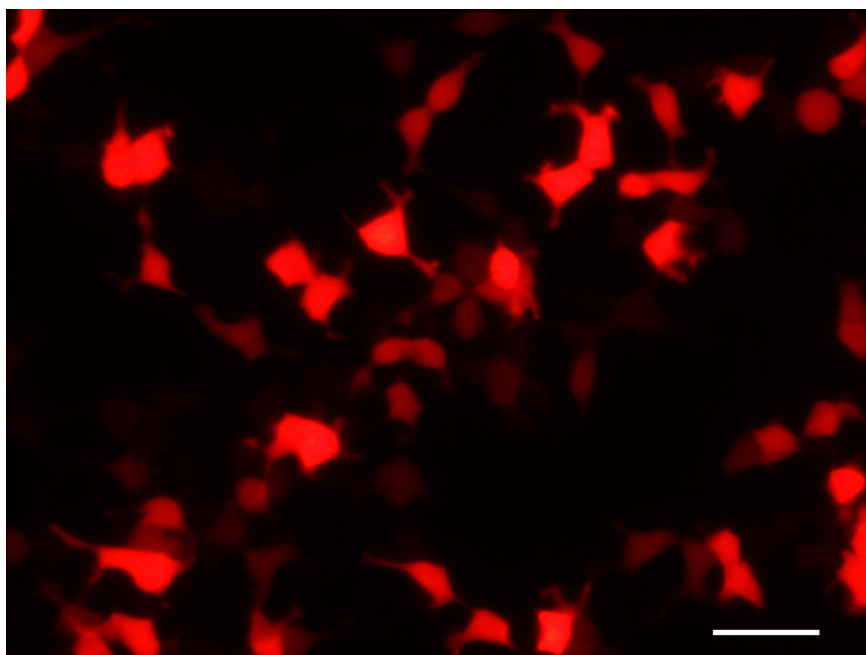

Fig. S1B

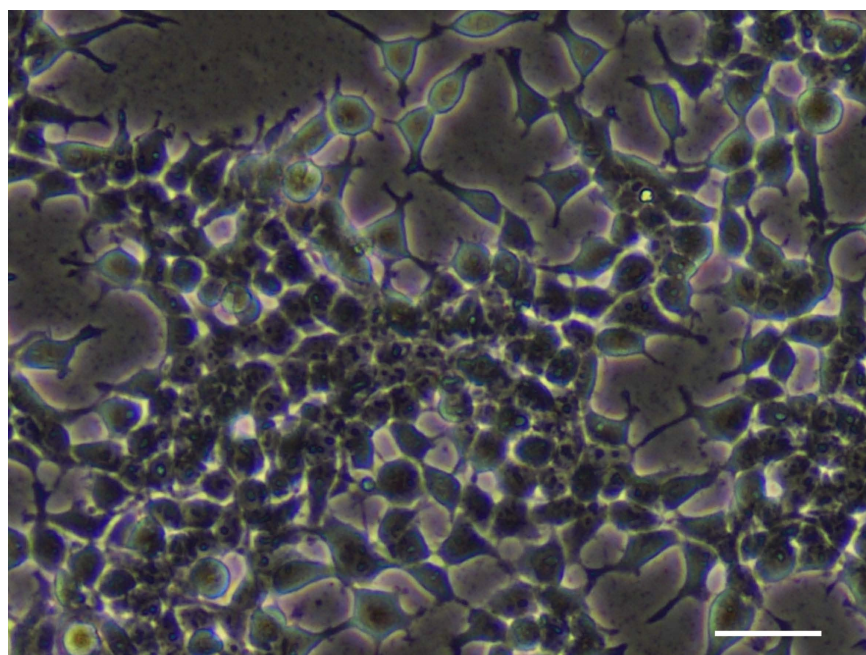

Supplement: Supplementary file 1 — Fig. S1. Transient transfection of dsRed in HEK‐293T cells. (A) dsRed expressed all over the cell; (B) in DIC. Scale bar = 50 μm. [file FEB4-9-1860-s001.pdf]
